# Supplementary material for: Dynamic changes in the suitable areas for the pinewood nematode in the Sichuan–Chongqing Region of China
Source: PLoS One. 2023 Oct 19;18(10):e0292893. doi: 10.1371/journal.pone.0292893 (PMC10586667; doi:10.1371/journal.pone.0292893)
Supplement: Attachment — Submitted filename: Research evaluation.docx [file pone.0292893.s001.docx]

**Research evaluation**

**Dynamic Changes of Suitable Areas for the Pinewood Nematode in Sichuan-Chongqing Region of China**

**Research Summary**

The idea of the research is concerned with an important topic in the field of Modeling of the Pinewood Nematode (PWN), to simulate the current and future geographic distribution of PWN in the Sichuan-Chongqing region of China based on the MaxEnt model, The results indicated the excellent prediction performances with the AUC of more than 0.9. The key factors selected were the altitude, the max temperature of warmest month, the annual precipitation, and the precipitation of wettest quarter and the min temperature of coldest month. The suitable distribution areas of the PWN in current and future conditions were mainly concentrated on metropolitan area and the northeast of Chongqing and the southeast and eastern of Sichuan Province. Most importantly, besides the actual distribution area, four newly discovered A, B, C and D, belong to the suitable distribution areas and should be strictly monitored for the PWN presence in recent years. To the period of 2050s, the suitable distribution areas of the PWN shows an increasing trend. Four newly discovered suitable distribution areas, for example, E, F, G and H, were identified as increasing suitable areas. the key factors identified were the altitude of ˂ 400 m, the max temperature of warmest month (bio05) of >37.5°C, the annual precipitation (bio12) of 1100~1250 mm, the precipitation of wettest quarter (bio16) of 460~530 mm and the min temperature of coldest month (bio06) of >4.0°C, respectively, indicating that the PWN is suitable for living in low altitude, warm and humid areas. Altogether, human activities concerned with pine trees and vector of the Japanese pine sawyer in Sichuan-Chongqing region should be intensively controlled to prevent the PWD from spreading to these newly discovered suitable areas.

**The conclusion** is good. It shows the importance of the study in terms of the suitable distribution areas of the PWN in current and future conditions were mainly concentrated on metropolitan area and the northeast of Chongqing and the southeast and eastern of Sichuan Province, and it is complete and clear.

**Keywords** are good and suitable for research.

**The introduction** is comprehensive and it deals with the subject of the study with a high professionalism and presents all the ideas related to the topic presented in the appropriate literature, where it referred to Pine wilt disease (PWD), mainly evoked by the pine wood nematode (PWN), is listed as one of the largest forestry alien pests for Pinus spp. worldwide.

The researchers pointed out the main problem the entire pine forest was completely destroyed in a few years once infested, which affirmatively causes huge economic, ecological and social losses in agriculture and forestry. In addition, The PWN being the causal agent of the PWD originating from North America, has been carried by way of the timber trade to Japan firstly, from where it has been spread into more than 40 countries, for instance South Korea, China in Asia and Germany, Scandinavia, France, Poland etc. in Europe. In China, the PWN has rapidly spread to 18 Provinces (Cities, Districts) and 588 counties (Cities, Districts) and destroyed 0.65 million hm2 of pine forests, and 60 million hm2 will be threatened by the PWN since it was first discovered in Nanjing, Jiangsu Province of China in 1982.

At present, few effective methods have been found to control its spread so far, so it is still spreading to other suitable areas along with growing trade and transportation, which attract the attention from the entire society. Consequently, it is necessary for local government to assess scientifically the potential hazards for the invasions of the PWN, and take some measures for preventing from its spreading. As a result, the spatial distribution of the PWN under current and future condition are required to assess as early as possible so that some eradication and prevention measures have been formulated in advance. Out of various SDMs, the MaxEnt (Maximum Entropy Modelling) model has been proved to be better relative to other SDMs. so the prediction results are rough to be difficult to layout scientific investigations and control pest infestations; ⑶lack of innovation in research methods, compared with our new method by using the newly introduced Maximum Youden Index and the average habitat suitability based on 10 replicates by cross-validation.

In this study, the current and future geographical distribution of the PWN was simulated by using the MaxEnt model by 421 known coordinates and 20 environmental layers in Sichuan-Chongqing region with 2 GCMs (Global climate models). The results of this study can help predict the determine the potentially suitable distribution of the PWN under current environmental condition; identify the increased and decreased suitable potential distribution to the period of 2050s; clarify some key factors which may limits its potential distributions, ultimately supplying objective measures for hindering this species from spreading.

**Materials and methods** Sample collection was good and clear, terms included Species distribution samples, Current environmental data, Filtering of environmental factors, Future environmental data, Predicting potential distribution, Model performance and influencing factors. The methods were presented in a precise and well detailed manner.

The results were well presented and consistent with the method of work, including the following main headings: Modeling evaluation and current geographic distribution, Environmental variable assessment and their threshold, Potential distribution pattern under future environmental condition, the results were good in the presentation method.

**Discussion paragraph**,

Insect hosts are of great importance in incubating, transporting and spreading Biological elements (Nematodes), so I ask: What is the role of the Biological insect vector, the Japanese pine sawyer (*Monochamus alternatus*)? What is the efficiency of transmission of Nematodes Pinewood nematode (PWN)?

It has been stated that this factor (the Biological insect vector, the Japanese pine sawyer (*Monochamus alternatus*)) is less important than human activity, and this is not clear enough. Therefore, he suggested studying this important factor the Japanese pine sawyer (*Monochamus alternatus*) in terms of its current and future places of spread and distribution, and introducing this factor as a basic element in improving the described equations, as well as the impact of human activity in this aspect.

**References** Well Attributed, Latest Reference 2022, I Found some minor errors in the formatting of the list of references, and corrected on the references.

In conclusion, I recommend publishing this research in your important and discreet journal due to its scientific importance, after making the required modifications.

**Latakia, 16/7/2023**

**Reviewer**

**Prof. Dr. Nabil ABO KAF**
