# Supplementary material for: Dynamic changes in the suitable areas for the pinewood nematode in the Sichuan–Chongqing Region of China
Source: PLoS One. 2023 Oct 19;18(10):e0292893. doi: 10.1371/journal.pone.0292893 (PMC10586667; doi:10.1371/journal.pone.0292893)
Supplement: Attachment — Submitted filename: revised_19bbc.doc [file pone.0292893.s002.doc]

**The discipline: Ecology**

The running header: Dynamic Changes of Suitable Areas of the pinewood nematode

**Dynamic Changes of Suitable Areas for the Pinewood Nematode in Sichuan-Chongqing Region of China**

Hongqun Li^1^, Xiaolong Peng^2^, Peng Jiang^2^, Ligang Xing^1^*, Xieping Sun^1^

1.School of modern agriculture and Bioengineering, Yangtze Normal University, Chongqing 408100, China;

2.Yan 'an Huanglongshan forestry bureau, Yan 'an 715700, China.

*Corresponding Author

Ligang Xing

School of modern agriculture and Bioengineering, Yangtze Normal University, Chongqing 408100, China

Telephone: +1-405-325-6076; E-mail: lihongqun2001@126.com

Journal to be submitted:

Nov 20, 2022

## Abstract: Pinewood nematode (PWN), one of the largest forestry alien pests in China, has resulted in a number of deaths of conifer forest in Europe and Asia and is spreading to other suitable areas worldwide. And some information on the spatial distribution of the PWN can provide important information for management of this species. Here the current and future geographical distribution of the PWN was simulated in Sichuan-Chongqing region of China based on MaxEnt model. The results indicated the excellent prediction performances with the AUC of more than 0.9. The key factors selected were the altitude, the max temperature of warmest month, the annual precipitation, the precipitation of wettest quarter and the min temperature of coldest month, with thresholds of ˂400 m, >37.5°C, 1100~1250 mm, 460~530 mm and >4.0°C, respectively, indicating that the PWN is suitable for living in low altitude, warm and humid areas. The suitable distribution of the PWN is mainly concentrated on metropolitan area and the northeast of Chongqing and the southeast and eastern of Sichuan Province. Most importantly, besides the actual distribution area, these newly identified A, B, C and D in recent years and increased E, F, G and H in the period of 2041-2060 (2050s) belong to the suitable distribution ranges yet and should be strictly monitored for the PWN presence. Altogether, the suitable distribution ranges of the PWN in Sichuan-Chongqing region shows an increasing trend**,** so human activities concerned with pine trees and vector of the Japanese pine sawyer should be intensively controlled to prevent the PWD from spreading to these newly discovered suitable areas.

**Key words:** Pinewood nematode; Climate change; the Maximum Youden Index; MaxEnt model

**INTRODUCTION**

Pine wilt disease (PWD), mainly evoked by the pine wood nematode (PWN), is listed as one of the largest forestry alien pests for *Pinus* spp. worldwide (Kim *et al*., 2020). This disease usually results in the mortality of the infected trees in about forty days because the PWN can reproduce quickly and destroy the vascular system of the entire tree once inside a pine tree (Robinet *et al*., 2011; Ye *et al*., 2019). Sometimes, the entire pine forest was completely destroyed in a few years once infested, which affirmatively causes huge economic, ecological and social losses in agriculture and forestry, so the PWD is jokingly called as the "cancer" or the "bird flu" of pine tree (Cheng *et al*., 2015). The PWN being the causal agent of the PWD originating from North America, has been carried by way of the timber trade to Japan firstly, from where it has been spread into more than 40 countries, for instance South Korea, China in Asia and Germany, Scandinavia, France, Poland etc. in Europe (Ju *et al*., 2010; Ikegami & Jenkins, 2018). In China, the PWN has rapidly spread to 18 Provinces (Cities, Districts) and 588 counties (Cities, Districts) and destroyed 0.65 million hm^2^ of pine forests, and 60 million hm^2^ will be threatened by the PWN since it was first discovered in Nanjing, Jiangsu Province of China in 1982 (Ye *et al*., 2019). At present, few effective methods have been found to control its spread so far, so it is still spreading to other suitable areas along with growing trade and transportation, which attract the attention from the entire society (Ju *et al*., 2010). Consequently, it is necessary for local government to assess scientifically the potential hazards for the invasions of the PWN, and take some measures for preventing from its spreading.

The species distribution models (SDMs) has been widely used in the protection of endangered species, the priority evaluation of reserve design, the diffusion of alien invasive species and so on (Wang *et al*., 2007; Xu *et al*., 2014). Habitat fragmentation and invasion of alien species are primary factors leading to biodiversity loss (Wang *et al*., 2019). For its spreading of the PWN, through completely analyzing, one of the important methods to stop alien organisms from causing harm to the invasive area is to control invasive species to enter into the suitable area for its survival (Robinet *et al*., 2011; Xu *et al*., 2014). Some researches have indicated that early control of alien species is more efficacious and affordable than prevention and control after invasion (Puth & Post, 2010; Li *et al*., 2022). As a result, the spatial distribution of the PWN under current and future condition are required to assess as early as possible so that some eradication and prevention measures have been formulated in advance. Out of various SDMs, the MaxEnt (Maximum Entropy Modelling) model has been proved to be better relative to other SDMs (Phillips *et al*., 2006; Qin *et al*., 2017). In the past decade of research, some scientists assessed the potential distribution of the PWN in Chongqing city or Sichuan Province alone by known distribution records together with layers of environmental variables (Wei *et al*., 2019; Li *et al*., 2022), but lack of similar research in Sichuan and Chongqing together. Therefore, these related studies are relatively incomplete, which are mainly reflected in: ⑴the relatively small area, because in the prediction of MaxEnt model, only in large scale can it show higher accuracy and large error in small scale, which originated from some facts that the expansion of spatial-scale means that more species information can be obtained (Zhuang *et al*., 2018; Ruan *et al*., 2022); ⑵lack of accurate location analysis to increase and decrease, so the prediction results are rough to be difficult to layout scientific investigations and control pest infestations; ⑶lack of innovation in research methods, compared with our new method by using the newly introduced Maximum Youden Index and the average habitat suitability based on 10 replicates by cross-validation (Liu *et al*., 2013; Xia *et al*., 2019); ⑷only one specific GCM (global climate model) to be difficult to explain related uncertainties owing to be lack of comparison of prediction results from multiple GCMs (Wei *et al*., 2019; Li *et al*., 2022); ⑸lack of multicollinearity analysis among environmental variables, which was regarded as error source (Jaryan *et al*., 2013; Jia *et al*., 2017), etc. Moreover, climate change in temperature and precipitation etc. will affect the geographical distribution of animals and plants (Qin *et al*., 2017), increase invasion of alien species and improve losses of biodiversity (Wang *et al*., 2019). And global warming is expected to increase the invasion of alien species through interfering with the structure and function of ecosystem and reducing its resistance to invasive species (Bethanya *et al*., 2010; Bellard *et al*., 2013; Vorsino *et al*., 2014). Here, the current and future geographical distribution of the PWN was simulated by using the MaxEnt model by 421 known coordinates and 20 environmental layers in Sichuan-Chongqing region with 2 GCMs (Global climate models). The aims are to ⑴ determine the potentially suitable distribution of the PWN under current environmental condition; ⑵ identify the increased and decreased suitable potential distribution to the period of 2050s; ⑶ clarify some key factors which may limits its potential distributions, ultimately supplying objective measures for hindering this species from spreading.

**MATERIALS AND METHODS**

**Study area**

The study area consists of Sichuan Province and Chongqing municipality of China, which are distributed between 95.917°–111.301°E and 25.271°–34.839°N in the southwest of China. In the past Chongqing belongs to Sichuan Province and then it was separated from Sichuan Province and established as the fourth municipality of China in June 18, 1997. Our study area includes main Hengduan and Daba mountain range, Chengdu plain and Chongqing hills of China. It has a mountainous subtropical humid climate here. The annual mean temperature varies from -10.5 to 22.2℃ and the annual mean precipitation from 429 to 1817 mm (Hijmans *et al*., 2005). In addition, the study area is suitable for pine trees to survive, especially for *Pinus massoniana* trees which are the main object of the PWN damage.

**Species distribution samples**

# The main distribution point data were obtained from three ways: ⑴the occurrence data of the PWN were collected from our team's field investigation in Chongqing by a GPS receiver in recent years. ⑵Some geo-names of existence points for this species were mainly acquired from Sichuan Forestry and grassland Bureau in 2019 (http://lcj.sc.gov.cn/scslyt/gsgg/2019) and ⑶Other geo-names provided by Chongqing Forest Disease and Pest Control Station of China, which only provide small place names where pine trees were infested by the PWN. Their coordinates were obtained by the Gaode map of China (https://www.amap.com/). In order to avoid spatial autocorrelation (Jaryan *et al*., 2013; Yang *et al*., 2020), duplicates points were deleted and only one existing point in each grid was retained, so a total of 421 existing points including 192 points in Chongqing and 229 ones in Sichuan Province were ultimately retained after checking their locations. Based on the above data, the distributional points of the PWN were saved in csv format on the basis of the demand of Maxent model.

**Current** **environmental data**

Anomalous changes in temperature and precipitation etc. have an influence on species’ geographical distribution ranges (Jia *et al*., 2017; Qin *et al*., 2017). To determine which environmental variables over the period of 1970–2000 most influence the distribution of the PWN, we selected 20 environment variables including 19 bioclimatic and one altitude variables in our model (Table 2) with a spatial resolution of approximately 1 km^2^, downloaded from global climate data (http://www.worldclim.org). Finally, 20 environmental variables were extracted by the boundary maps of Sichuan-Chongqing region from the above global raster data and transformed into asc pattern in ArcGIS 10.2. Additionally, China vector map was acquired from free spatial data of diva-gis (http://swww.diva-gis.org/Data).

**Filtering of environmental factors**

Because high multicollinearity among the environmental variables has been thought as error source (Jaryan *et al*., 2013; Jia *et al*., 2017), so to overcome this multicollinearity we extracted values from all 20 environmental variables combined with 421 presence points in ArcGIS 10.3 and perform relevant analysis. Based on Pearson correlation coefficient (/r/ ≥ 0.8) in SPSS 19.0 and taking into consideration the importance of each environmental variables devoted to predictor contributions, we excluded several variables which made a small contribution to the model and only retained those that made a large contribution to the model. Finally, a total of remaining 8 variables were retained and utilized for modelling (Table 2). Meanwhile, to meet the needs of this Maxent model, all these remaining variables were converted to asc formats.

**Future environmental data**

The RCPs (Representative Concentration Pathways), more scientific and near to the real climate change owing to considering the impact of various strategies to deal with climate change on future greenhouse gas emissions (Wang *et al*., 2017), were announced by the IPCC (Intergovernmental Panel on Climate Change) in the Fifth IPCC Assessment Report (AR5). Furthermore, the scenarios of the RCPs at 30 arc-second resolution, free from the WorldClim database in [2050](https://www.worldclim.org/data/v1.4/cmip5_30s.html#2050) (average for 2041-2060) (https://www.worldclim.org/data/v1.4/cmip5_30s.html) (Hijmans *et al*., 2010), include four future [representative concentration pathways](http://en.wikipedia.org/wiki/Representative_Concentration_Pathways) such as RCP2.6, RCP4.5, RCP6.0 and RCP8.5, which represents greenhouse gas emissions with radiative forcing values of 2.6 /4.5/6.0/8.5 w/m^2^ in the year of 2100. In this study, this future geographic distribution ranges of the PWN were simulated by using two GCMs (Global climate models such as BCC-CSM1.1 and GISS-E2-R) for three RCPs expressing low (RCP2.6), middle (RCP4.5) and high (RCP8.5) greenhouse gas emission over the period of 2050s, widely used in previous studies (Karspeck *et al*., 2015; Bosso *et al*., 2017). According to our hypothesis in advance, one elevation variable was remained unchanged under different environmental conditions. Finally, a total of 8 environmental variables similar to those data in current condition, were imported directly into the MaxEnt model in future conditions.

**Predicting potential distribution**

The current and future geographical distributions of the PWN were simulated based on the MaxEnt model (Version 3.4.1, [http://www.cs.princeton.edu/∼schapire/maxent/), mainly because the model performs better than do with other SDMs (Qin *et al*., 2017; Wang *et al*., 2017).](http://biodiversityinformatics.amnh.org/open_source/maxent/).) The MaxEnt model was used to predict species’ geographical distribution based on presence-only data along with layers of surrounding variables according to the maximum entropy theory (Phillips *et al*., 2006). MaxEnt model incorporates interactions between the variables and can deal with categorical and continuous environmental variables (Khanum *et al*., 2013; Xu *et al*. 2013). In addition, the demand for computer configuration is lower, the operation is more convenient and its predicted results are more stable (Li *et al*., 2022). When modeling, 75% of all occurrence data were randomly chosen to train the model, and the remaining 25% were utilized for testing (Wang *et al*., 2010; Qin *et al*., 2017). Simultaneously, “Do jackknife to measure variables importance” and " Create response curves” commands were also checked with a tick in the model interface with default settings for other parameters because these default setting is enough to ensure better the models’ effectiveness (Kumar *et al*., 2014). Moreover, to ensure the steadiness of the results, the model was run 10 replicates by cross-validation and the average habitat suitability was considered as the final result in logistic format and asc types (Khanum *et al*., 2013; Zhang *et al*., 2020). To display and further analyses, this final result was converted to raster format in ASCⅡ to Raster of ArcGIS 10.2 and cell values of the predicted map ranges from 0 (the lowest habitat quality) to 1 (the highest habitat quality) for this species. Owing to the need of binary maps, the continuous suitability index maps were converted into suitable and unsuitable areas according to the Maximum Youden Index (Liu *et al*, 2013; Xia *et al*., 2019). The Maximum Youden Index (specificity+sensitivity-1) was usually used to be the cutoff point, which is advantage over other threshold value in transforming the continuous area into ‘suitable area’ and ‘unsuitable area’ (Liu *et al*, 2013). And according to the past literature (Li *et al*., 2022), the suitable area is assigned as 1 and the unsuitable one is 2 under current condition, while in future conditions the suitable is assigned a value of 3 and the unsuitable is a value of 4. Then, two sets of data from the current and future conditions multiplied in ArcGIS 10.2, so that the cell value of 3 indicates the unsuitable area and 8 signifies the suitable one for this species, while 4 indicates the increased suitable area, 6 signifies the decreased suitable one in future conditions. Finally, habitat areas are computed based on properly project coordinates.

**Model performance and influencing factors**

The prediction performances were assessed by the AUC value equal to the area under the receiver operating characteristic curve acquired directly from the analysis of MaxEnt model and nowadays this method was widely utilized in the evaluation of predictive powers for many models and considered as the best evaluation index (Qin *et al*., 2017; Wang *et al*., 2010; Wang *et al*., 2021). The AUC value was from 0.5 to 1.0 (Qin *et al*., 2017). An AUC value equal to 0.50 implies that the prediction performance was not better than that of the random model, whereas a value equal to 1.0 shows the best performance (Jaryan *et al*., 2013; Xu *et al*., 2014). Specifically speaking, an AUC values of less than 0.5 shows that the performance is worse than that of the random one, 0.5~0.7 indicates low performance, 0.7~0.9 signifies moderate performance, and more than 0.9 suggests excellent performance (Kumar *et al*., 2014; Hu *et al*., 2015). Finally, based on the predictor contributions, permutation importance and regularized training gain, we can be able to estimates the relative influence of individual predictors to the species’ habitat suitability (Dong *et al*., 2019; Zhang *et al*., 2020; Wang *et al*., 2021). In addition, Maxent-generated response curve was produced automatically by Maxent model to analyze the relations between environmental variables and the probability of occurrence (Hu *et al*., 2015).

**RESULT**

**Modeling evaluation and current geographic distribution**

In this study, all the average AUC of model training and testing are more than 0.9 for the prediction performances under climate change conditions (Table 1). These results indicated that all the prediction performances are excellent for prediction of this species. Based on the Maximum Youden Index (0.202), the final potential distribution map were reclassified into the suitable and unsuitable areas. The suitable geographic distribution of the PWN in the study region mainly concentrated on metropolitan area and the northeast of Chongqing and the southeast and eastern of Sichuan Province, among them, except for newly discovered A, B, C and D (Figure 1), the other suitable distribution areas were covered by lots of distribution points, implying that the result of the Maxent model predicting the presence of the PWN were consistent with the actual distribution area. Most importantly, these newly discovered A (Guangan, Yingshan, Pengan, Yilong, Bazhong and Pingchang), B (Longchang, south of Neijiang and Rongxian, southeast Weiyuan, west Luxian and Rongchang of Chongqing), C (Yubei, Beibei, southeast Hechuan, Shapingba, Jiangbei, Nanan and Bishan) and D (Dianjiang and Liangping) belong to the suitable distribution areas predicted by the Maxent model yet and should be strictly monitored even if few occurrence points were found in these places. The analysis showed yet that the suitable and unsuitable areas occupied 12.68% and 87.32% of the whole study area, respectively, and the suitable and unsuitable areas in Sichuan Province accounted for 8.23% and 91.77% whereas in Chongqing they only amounted to 38.90% and 61.10%, respectively.

**Table 1 The modeling prediction precision of AUC in the periods of 1970-2000 and 2041-2060 (2050s)**

| GCMs | Periods | Training AUC | Test AUC | AUC of Random prediction |
| --- | --- | --- | --- | --- |
| Current | 1970-2000 | 0.9403±0.0036 | 0.9414±0.0071 | 0.5 |
| BCC-CSM1.1-rcp2.6 | 2050s^a^ | 0.9364±0.0012 | 0.9307±0.0149 | 0.5 |
| BCC-CSM1.1-rcp4.5 |  | 0.9349±0.0016 | 0.9293±0.0191 | 0.5 |
| BCC-CSM1.1-rcp8.5 |  | 0.9338±0.0007 | 0.9283±0.0090 | 0.5 |
| GISS-E2-R-rcp2.6 |  | 0.9368±0.0012 | 0.9321±0.0141 | 0.5 |
| GISS-E2-R-rcp4.5 |  | 0.9407±0.0011 | 0.9361±0.0110 | 0.5 |
| GISS-E2-R-rcp8.5 |  | 0.9369±0.0012 | 0.9310±0.0122 | 0.5 |

^a^2050s = average for the year of 2041-2060

**Environmental variable assessment and their threshold**

Among 8 environmental variables, based on percent contributions, the altitude variable had the highest score (Table 2), indicating that this variable significantly has an effect on the distribution of the PWN under the current condition; and then followed by the max temperature of warmest month (bio05), with 34.9%. The cumulative contributions of these two parameters reached to 70.8%. Meanwhile, permutation importance is the value of each environmental variable randomly replaced on the training existence and background data. And the larger the value is, the greater the dependence of the model on this variable (Zhang *et al*., 2020). Therefore, the top four environmental variables in the value of permutation importance are the max temperature of warmest month (bio05), the annual precipitation (bio12), the min temperature of coldest month (bio06) and the precipitation of wettest quarter (bio16). The cumulative permutation importance of these four parameters amounted to 80.2%. By the jackknife test (Figure 2), the max temperature of hottest month (bio-05), the min temperature of coldest month (bio-06) and the altitude were the top three contributions to the distribution of the PWN relative to other environmental variables. On the whole, these five main factors affecting the potential geographical distribution of the PWN are: the altitude, the max temperature of warmest month (bio05), the annual precipitation (bio12), the precipitation of wettest quarter (bio16) and the min temperature of coldest month (bio06).

To further clarify the thresholds of main environmental variables under the current condition and eliminate the correlations between the above-mentioned key factors, these five environmental variables are individually imported into MaxEnt model for modeling and plotting response curve of probability and environmental variables. The results indicate that the threshold value of these five key variables (probability of presence >0.5) were: the altitude variable ˂ 400 m, the max temperature of warmest month (bio05) >37.5°C, the annual precipitation (bio12) from 1100 to 1250 mm, the min temperature of coldest month (bio06) >4.0°C and the precipitation of wettest quarter (bio16) from 460 to 530 mm (Figures are not shown).

**Table 2 The environmental factors used in this study, their contribution and permutation importance under current environmental condition**

| Code | Description | Percent contribution | Permutation importance | Code | Description | Percent contribution | Permutation importance |
| --- | --- | --- | --- | --- | --- | --- | --- |
| Alt | **Altitude** | **35.9** | 1.0 | bio06 | **Min temperature of coldest month** | 4.6 | **14.9** |
| bio05 | **Max temperature of warmest month** | **34.9** | **36.3** | bio12 | **Annual precipitation** | 2.7 | **17.5** |
| bio14 | Precipitation of driest month | 12.8 | 6.0 | bio09 | Mean temperature of driest quarter | 2.2 | 9.5 |
| bio03 | Isothermality | 4.9 | 3.2 | bio16 | **Precipitation of wettest quarter** | 2.1 | **11.5** |

The highlighted variables, selected through their contribution and permutation importance, were five main influencing factors.

**Potential distribution pattern under future environmental condition**

According to the Maximum Youden Index from different GCMs (Table 3), the final potential distribution map were classified into the suitable and unsuitable areas. To the period of 2050s, model results show similar distribution areas in the suitable geographic distribution of the PWN in Sichuan-Chongqing region of China, however, compared with current suitable distribution areas, local areas show an increasing or decreasing trend in the future. Taken altogether, the suitable distribution areas of the PWN in the study area shows an increasing trend too because the increased area is greater than the reduced area (Table 3). More importantly, in the period of 2050s four newly discovered suitable distribution areas, for example, E (Langzhong, east Nanbu, northeast Nanchong and southeast Changxi), F (southeast Zizhong, middle of Neijiang, and northwest Rongchang of Chongqing), G (noreteast Yaan, southweat Mingshan, northeast Hongya, middle of Jiajiang and northeast Leshan) and H (Luxian, west Hanjiang, southwest Yongchuan and southeast Dazu of Chongqing), were identified as increasing suitable areas by the Maxent model, indicating that the suitable habitat areas in Sichuan-Chongqing regions were predicted to be increased gradually to 1.26~2.58% in BCC-CSM1.1 and 0.72~3.44% in GISS-E2-R to the period of 2050s (Table 3). Consequently, in Sichuan-Chongqing region of China we should strictly monitor four newly discovered suitable areas in the future (Figure 3).

**Table 3 Changes of the suitable habitat for *B. xylophilus* to the period of 2041-2060 (2050s)**

| Climate scenarios | GCMs | Maximum Youden index | Decreased suitable habitat | | Increased suitable habitat | | Total habitat change | |
| --- | --- | --- | --- | --- | --- | --- | --- | --- |
|  |  |  | Area/km^2^ | Percent/% | Area/km^2^ | Percent/% | Area/km^2^ | Percent/% |
| RCP2.6 | BCC-CSM1.1 | 0.1765 | 1585.65 | 0.26 | 17219.20 | 2.84 | 15633.55 | 2.58 |
|  | GISS-E2-R | 0.1647 | 2439.15 | 0.40 | 11045.60 | 1.82 | 8606.45 | 1.42 |
| RCP4.5 | BCC-CSM1.1 | 0.1827 | 3325.98 | 0.55 | 10978.2 | 1.81 | 7652.22 | 1.26 |
|  | GISS-E2-R | 0.1603 | 3998.63 | 0.66 | 8357.38 | 1.38 | 4358.75 | 0.72 |
| RCP8.5 | BCC-CSM1.1 | 0.1693 | 2304.31 | 0.38 | 15095.8 | 2.49 | 12791.49 | 2.11 |
|  | GISS-E2-R | 0.1920 | 1662.59 | 0.27 | 22522.7 | 3.71 | 20860.11 | 3.44 |

**DISCUSSION**

The PWD, leaded to by the PWN (*B. xylophilus*), as one of the greatest threats to pine trees and being spreading all over the world, has leaded to the tremendous economic, ecological and biodiversity losses to the intruding area in more than 40 countries (Ju *et al*., 2010). To date, few effective and economical methods have been found to control its spreading, so it is still spreading to other suitable habitats. Moreover, very little is known about concrete information on the direction that the PWN expanded to in Sichuan-Chongqing region and its main affecting factors that limit the geographic distribution of the species. Recently, with the development of biostatistics and applied ecology, the SDMs, especially the ecological models, such as Gam, Cart, GARP and MaxEnt etc. can provide powerful models for predicting the geographic distribution of the species with only the data of animal appearance points to get satisfactory results (Wang *et al.*, 2007; Wang *et al.*, 2010). Out of them, the MaxEnt model can consistently behaves better with less sampled data compared with other niche models and be widely utilized for estimating the geographic distribution of suitable area for species in in many field (Khanum *et al*., 2013; Wang *et al*., 2017). In the past, our team predicted the potential distribution of the PWN in Chongqing municipality of China by MaxEnt model in climate change conditions (Li *et al*., 2022). Nevertheless, owing to be lack of accurate location analysis to increase and decrease, so the prediction results are rough to be difficult to prevent and control the PWN accurately. In the present study, these models could provide satisfactory results for this species with the average AUC of model training and testing >0.9, implying to be excellent performance under current and future environmental conditions. As shown in Figure 1, in current condition, the ideal distribution area of the PWN in the study region is mainly concentrated on metropolitan area and the northeast of Chongqing municipality and the southeast and eastern of Sichuan Province (Figure 1), which is consistent with the suitable distribution area in the period of 2041-2060 (2050s), implying that from now to 2050s, above these suitable areas should be strictly monitored. Most importantly, four newly suitable distribution areas, such as A, B, C and D, were identified and should be strictly monitored in recent years, because sporadic points also appear here (Figure 1) while in the period of 2050s, through the newly introduced Maximum Youden Index (Liu *et al*, 2013; Xia *et al*., 2019), other four newly identified suitable distribution areas, for example, E, F, G and H, were identified distinctly as increasing suitable areas (Figure 2) and should be strictly monitored yet. In summary，these maps may be utilized to layout field investigations and project in more detail from now to 2050s with the limited research funds and manual labors. In addition, between pine trees, spread of the PWN with almost no diffusion ability by itself, depends on insect vector, i. e, the Japanese pine sawyer (*Monochamus alternatus*), being the most important carrier of the PWN over relatively short distances and the human-mediated activities in the spread of long-distance (Robinet *et al*., 2011; Chen *et al*., 2018). As a consequence, except for these newly discovered suitable areas, original suitable area in red should be strictly managed, e.g. some pitfalls of catching the insect vector, burning trees with symptoms of the PWD, prohibiting conveyance of infested lumber or packaging, especially relating to pine trees, etc. to prevent the PWN from entering these newly discovered suitable areas.

The geographical distribution of species was mainly affected by temperature, rainfall, terrain and so on (Rehfeldt *et al*., 1999; Jia *et al*., 2017). In the past, Robinet et al. (2009) had found that the key variables identified were July mean temperature（T_Jul_≥21.3℃）and January mean temperature（T_Jan_≥-10℃）, which markedly affect the potential distribution of the PWN. In the present study, these five main factors affecting the potential geographical distribution of the PWN are: the altitude of ˂ 400 m, the max temperature of warmest month (bio05) of >37.5°C, the annual precipitation (bio12) of 1100~1250 mm, the precipitation of wettest quarter (bio16) of 460~530 mm and the min temperature of coldest month (bio06), with thresholds of >4.0°C, respectively. These results are almost similar to those studies in the past (Robinet *et al*., 2009; Cheng *et al*., 2015; Li *et al*., 2022). The suitable habitat of the PWN mainly was concentrated in low altitude areas (˂400 m), which supports the fact that no pine wood nematode has been found in the high-altitude areas of Sichuan Province adjacent to Tibet (Fig 1). The reason may be that human-beings participate in pine-related activities frequently, for instance the transportation of infected wood and packaging boxes, especially those related to infected pine trees, which support past views that human-related diffusion plays an important role in the spread of the PWN (Robinet *et al*., 2009). The Max temperature of warmest month with thresholds of >37.5°C is closely related to the Japanese pine sawyer, because it belongs to typical tropical and subtropical groups (Wang,1988), and facilitate its spread far away (Ju *et al*., 2010; Li *et al*., 2022). The annual precipitation (bio12), the precipitation of wettest quarter (bio16) and the min temperature of coldest month (bio06) with thresholds of 1100~1250 mm, 460 ~530 mm and >4.0°C indicated that the PWN is suitable for living in warm and humid areas, which may improve the eclosion rate of the Japanese pine sawyer being the main carrier of the PWN. This is the same view as in the past (Robinet *et al*., 2009; Cheng *et al*., 2015; Wei *et al*., 2019). The min temperature threshold of coldest month (bio06) is more than 4℃. When the minimum temperature is below 4℃, pine trees will grow weak and its resistance declines, once the PWN is transmitted to this host trees by the Japanese pine sawyer, it is easy for this tree to be infected and this disease occurs (Wei *et al*., 2019). Besides, the –10℃ January mean air temperature isotherm is advised as the northern limit for the geographic distribution of the Japanese pine sawyer in China (Ma *et al*., 2006). The situation supports previous facts that the number of the Japanese pine sawyer increased after the occurrence of ice and snow disasters in southeast China (Dai *et al*., 2010).

**CONCLUSION**

⑴ The suitable distribution areas of the PWN in current and future conditions were mainly concentrated on metropolitan area and the northeast of Chongqing and the southeast and eastern of Sichuan Province. Most importantly, besides the actual distribution area, four newly discovered A, B, C and D, belong to the suitable distribution areas and should be strictly monitored for the PWN presence in recent years. ⑵ to the period of 2050s, the suitable distribution areas of the PWN shows an increasing trend. Four newly discovered suitable distribution areas, for example, E, F, G and H, were identified as increasing suitable areas. ⑶ the key factors identified were the altitude of ˂ 400 m, the max temperature of warmest month (bio05) of >37.5°C, the annual precipitation (bio12) of 1100~1250 mm, the precipitation of wettest quarter (bio16) of 460~530 mm and the min temperature of coldest month (bio06) of >4.0°C, respectively, indicating that the PWN is suitable for living in low altitude, warm and humid areas. Altogether, human activities concerned with pine trees and vector of the Japanese pine sawyer in Sichuan-Chongqing region should be intensively controlled to prevent the PWD from spreading to these newly discovered suitable areas.

**Acknowledgements.** We are particularly helpful to Chongqing Forest Disease and Pest Control Station for the assistance in data collection. We are particularly grateful to Simeng Zhang for the support assistance in data collation. Two anonymous referees were especially thanked for their critical suggestions which heightened this manuscript quality. The work was financially subsidized by the National Natural Science Foundation of China, grant number 31870515; Excellent Achievement Transformation Project in Universities of Chongqing, grant number KJZH17132.

**Conflict of interest disclosure**. The authors declare that there is no conflict of interest.

**Ethical standards**. Not applicable.

**References:**

**Bellard C, Thuiller W, Leroy B, Genovesi P, Bakkenes M and Courchamp F(2013)** Will climate change promote future invasions?. *Global Change Biology*, 19: 3740-3748.

**Bethanya B, Davids W and Michael O** (2010) Climate change increases risk of plant invasion in the Eastern United States. [*Biological Invasions*](http://www.baidu.com/link?url=e2vAB48IFEU02p5oM8jtfR_b3chfs2vlIC7vBDDf4sqvD4xbeNbkUoL5UA9n93emARIcrw6xd3ZpNvoF6x2y97-jvGob4Q_Lhn4iLsZD1vW&wd=&eqid=f900c7390008d25100000006617ba9b4) 12(6), 1855-1872.

**Bosso L, Luchi N, Maresi G, Cristinzio G, Smeraldo S and Russo D** (2017) Predicting current and future disease outbreaks of *Diplodia sapinea* shoot blight in Italy: species distribution models as a tool for forest management planning. *Forest Ecology and Management* **400**, 655-664.

**Chen J, Cheng J and Lin T** (2018) Analysis of potential molecular targets in *Monochamus alternatus* (Coleoptera: Cerambycidae) inoculated with *Beauveria bassiana* (Deuteromycotina: Hyphomycetes). *Journal of Entomological Science* **53**(4), 533-542.

**Cheng G, Lu Q, Feng YM, Li YX, Wang YL and Zhang XY** (2015) Temporal and spatial dynamic pattern of pine wilt disease distribution in China predicted under climate change scenario. *Scientia Silvae Sinicae* **51**, 119-126.

**Dai LX, Liu YJ and Zhang Q** (2010) Influence of ice and snow disasters on the population of *Monochamus alternatus* and its counter-measures. *Forest Pest and Disease* **29**, 32-34.

**Dong X, Chu YMR, Gu XD, Huang QY, Zhang JD and Bai WK** (2019) Suitable habitat prediction of sichuan snub-nosed monkeys (*Rhinopithecus roxellana*) and its implications for conservation in baihe nature reserve, Sichuan, China. *Environmental Science and Pollution Research* **26**(31), 32374-32384.

**Hijmans RJ and Graham CH** (2010) The ability of climate envelope models to predict the effect of climate change on species distributions. *Global Change Biology* **12**(12), 2272-2281.

**Hijmans RJ, Cameron SE, Parra JL, Jones PG and Jarvis A** (2005) Very high resolution interpolated climate surfaces for global land areas. *International journal of climatology* **25**, 1965-1978.

**Hu XG, Jin Y, Wang XR, Mao JF and Li Y** (2015) Predicting impacts of future climate change on the distribution of the widespread conifer *Platycladus orientalis*. *Plos One* **10**, e0132326.

**Ikegami M and Jenkins T** (2018) Estimate global risks of a forest disease under current and

future climates using species distribution model and simple thermal model – pine wilt disease as a model case. *Remote sensing of environment* 409, 343-352.

**Jaryan V, Datta A, Uniyal SK, Kumar A, Gupta RC and Singh RD** (2013) Modelling potential distribution of *Sapium sebiferum*-an invasive tree species in western Himalaya. *Current Science* **105**, 1282-1288.

**Jia X, Ma FF, Zhou WM, Zhou L, Yu DB and Qin J** (2017) Impacts of climate change on the potential geographical distribution of broadleaved Korean pine (*Pinus koraiensis*) forests. *Acta Ecologica Sinica* **37**, 464-473.

**Ju YW, Li MY and Wu WH** (2010) Predictive methods of pine wilt disease in Jiangsu Province. *Scientia Silvae Sinicae* 46, 91-96.

**Karspeck A, Yeager S, Danabasoglu G and Teng H** (2015) An evaluation of experimental decadal predictions using CCSM4. *Climate Dynamics* 44, 907-923.

**Kim BN, Ji HK, Ahn JY, Kim S and Min J** (2020) A short review of the pinewood nematode, *Bursaphelenchus xylophilus*. *Toxicology and Environmental Health Sciences* 12(4), 1-8.

**Khanum R, Mumtaz AS and Kumar S** (2013). Predicting impacts of climate change on medicinal asclepiads of pakistan using maxent modeling. *Acta Oecologica* 49, 23-31.

**Kumar S, Graham J, West AM and Evangelista PH (**2014**)** Using district-level occurrences in Maxent for predicting the invasion potential of an exotic insect pest in india. *Computers and Electronics in Agriculture* **103**, 55-62.

**Li HQ**, **Xing LG, Liu XL, Pu YL, Yang YQ and Fu YY** (2022 a) Potential Impact of Climate Change on the Distribution of the Pinewood Nematode *Bursaphelenchus xylophilus* in Chongqing, China. *Pakistan Journal of Zoology* **54**, 809-816.

**Li HQ, Xing LG and Sun XP (2022 b)** Predicting the potential distribution of *Taxus wallichiana* var. *mairei* under climate change in China using Maxent modeling. *Pakistan Journal of Botany* **54**(4), 1305-1310.

**Liu C, White M and Newell G** (2013) Selecting thresholds for the prediction of species occurrence with presence-only data. [*Journal of Biogeography*](http://www.bioone.org/servlet/linkout?suffix=bibr31&dbid=16&doi=10.3161/068.042.0203&key=10.2307/2845564) **40**(4), 778-789.

**Ma RY, Hao SG, Kong WN, Sun JH and Kang L** (2006). Cold hardiness as a factor for assessing the potential distribution of the japanese pine sawyer *Monochamus alternatus* (Coleoptera: Cerambycidae) in China. *Annals of Forest Science* **63**(5), 449-456.

**Phillips SJ, Anderson RP and Schapire RE**（2006）Maximum Entropy Modelling of Species Geographic Distributions. *Ecological Engineering* **190**, 231-259.

**Puth LM and Post DM** (2010) Studying invasion: have we missed the boat? *Ecology Letter* 8, 715-721.

**Qin A, Liu B, Guo Q, Bussmann RW and Pei S**（2017）Maxent modeling for predicting impacts of climate change on the potential distribution of *Thuja sutchuenensis* Franch. an extremely endangered conifer from southwestern China. *Global* [*Conservation Ecology*](http://www.baidu.com/link?url=DXWCyNjWnu6hDm33xf7qI5YiIMdME_FS3B0xmxc7M-d8LdIFb3tbZpOo3uCtElnf) **10**, 139-146.

**Rehfeldt GE, Ying CC, Spittlehouse DL and Hamilton DA** (1999) Genetic responses to climate in pinus contorta: niche breadth, climate change, and reforestation. *Ecological Monographs* **69**(3), 375-407.

**Robinet C, Roques A, Pan HY, Fang GF, Ye JR., Zhang YZ and Sun JH** (2009) Role of human-mediated dispersal in the spread of the pinewood nematode in China. *PLoS One* **4**, e4646.

**Robinet C, Opstal NV, Baker R and Roques A** (2011) Applying a spread model to identify the entry points from which the pine wood nematode, the vector of pine wilt disease, would spread most rapidly across Europe. [*Biological Invasions*](http://www.baidu.com/link?url=e2vAB48IFEU02p5oM8jtfR_b3chfs2vlIC7vBDDf4sqvD4xbeNbkUoL5UA9n93emARIcrw6xd3ZpNvoF6x2y97-jvGob4Q_Lhn4iLsZD1vW&wd=&eqid=f900c7390008d25100000006617ba9b4) **13**, 2981-2995.

[**Ruan O,**](https://www.ecologica.cn/) **Liu SH, Chen F, Luo J and Hu HT (2022)** Habitat suitability evaluation of black-necked cranes based on multi-source remote sensing in Caohai National Nature Reserve, Guizhou. *Acta Ecologica Sinica* **42**(5), 1947-1957.

**Vorsino AE, Fortini LB, Amidon FA, Miller SE and Koob GA** (2014). Modeling hawaiian ecosystem degradation due to invasive plants under current and future climates. *PloS One* **9**, e102400.

**Wang AJ, Lu DY, Zhang GS, Huang HG, Wang Y, Hu SL, Ao M** (2021) Potential Distribution of Juniperus sabina under Climate Change in Eurasia Continent Based on MaxEnt Model. *Scientia Silvae Sinicae* **57**(8), 43-55.

**Wang CJ, Wan JZ and Zhang ZX** (2019). Will global climate change facilitate plant invasions in conservation areas? *Pakistan Journal of Botany* 51(4): 1395-1403.

**Wang Q** (1988). The Biogeography of *Monochamus* Guer (Coleoptera: Cerambycidae). *Scientia Silvae Sinicae* 24(3) , 297-304.

**Wang RL, Li Q, Feng CH, Shi CP (**2017**)** Predicting potential ecological distribution of *Locusta migratoria tibetensis* in China using MaxEnt ecological niche modeling. *Acta Ecologica Sinica* **37**(24), 8556-8566.

**Wang XY, Huang XL, Jiang LY, Qiao GX (**2010**)** Predicting potential distribution of chestnut phylloxerid (Hemiptera: Phylloxeridae) based on Garp and Maxent ecological niche models. *Journal of applied entomology* **134**, 45-54.

**Wang YS, Xie BY, Wan FH, Xiao QM, Dai LY** (2007) Application of ROC Curve Analysis in Evaluating the Performance of Alien Species’ Potential Distribution Models. *Biodiversity Science* **15**(4), 356-372.

**Wei ST, Li T and Lin YC** (2019) Prediction of the Potential Distribution of *Bursaphelenchus xylophilus* in Sichuan Province Using MaxEnt Model. *Sichuan Journal of Zoology* **38**(1), 37-46.

**Xia SS, Hu DM, Deng Y, Zhong X, Zhou CQ** (2019). Habitat partitioning between sympatric golden pheasant and temminck's tragopan at different spatial scales. *Acta Ecologica Sinica*, **39**(5) : 1627-1638..

### Xu Z, Peng H, Feng Z and Abdulsalih N (2014) Predicting current and future invasion of *Solidago canadensis* : a study from China. [**Polish Journal of Ecology**](http://www.baidu.com/link?url=n3NZut1QeO309AmjyWjpzXuJjKafbNi0jwvfV7GVGPXQjc2KoZmJgNUIxLZV7gO66A0BvvWaYw12_KXswcW-eDk4ybXRwXrW7muP_zPcXH9_wTEKln0OfePT22pee5uixMr1DFxbXBG4z5CMgpgjFJnDI0IB4z_VriFouYwPdJHQDmA6EDD6SYzAi7nFhIGsXPJrNe-vgCBdNLnjBcHYKssNkDxePzGhwRQSBSRmEVHzanAKvoPNQkKvRIe0PnFByEArVscyjYyq8bcNZFABHPUq__WZ0DiZ7AFdYr3dCHS) 62, 263-271.

**Yang N, Ma DY, Zhong X, Yang K, Zhou ZQ, Zhou HL and Zhou** **CQ** (2020) Habitat Suitability Assessment of Blue Eared-Pheasant Based on MaxEnt Modeling in Wanglang National Nature Reserve, Sichuan Province. *Acta Ecologica Sinica* **40**(19), 7064-7072.

**Ye JR** (2019) Epidemic status of pine wilt disease in China and its prevention and control techniques and counter measures. *Scientia Silvae Sinicae* **55**(9), 1-10.

**Yuan Y, Zhao XL, Chen ZY, Liu X, Lv J and Li H** (2012) Analyses on change characteristics of average temperature in Sichuan in 50 years. *Journal of Anhui Agricultural Sciences* **40**(4), 2251-2254.

[**Zhang H**](http://www.ecologica.cn/)**,** [**Zhao HX**](http://www.ecologica.cn/)**,** [**Wang H**](http://www.ecologica.cn/) (2020) Potential geographical distribution of *Populus euphratica* in China under future climate change scenarios based on Maxent model. *Acta Ecologica Sinica* **40**(18), 6552-6563.

**Zhuang HF, Zhang YB, Wang W, Ren YH, Liu FZ, Du JH and Zhou Y** (2018). Optimized hot spot analysis for probability of species distribution under different spatial scales based on Maxent model: manglietia insignis case. *Biodiversity Science* 2018, **26** (9), 931-940.

**Table 1 The modeling prediction precision of AUC in the periods of 1970-2000 and 2041-2060 (2050s)**

**Table 2 The environmental factors used in this study, their contribution and permutation importance under current environmental condition**

**Table 3 Changes of the suitable habitat for *B. xylophilus* to the period of 2041-2060 (2050s)**

**Figure 1 The potential geographic distribution of *B. xylophilus* in the periods of 1970-2000**

**Figure 2 Jackknife test for assessing the weight of each environmental variables on the geospatial distribution of *B. xylophilus* under the current condition**

**Figure 3** **The decreased and increased areas of suitable potential distribution for *B. xylophilus* to the period of 2050s**
