# Supplementary material for: Dynamic changes in the suitable areas for the pinewood nematode in the Sichuan–Chongqing Region of China
Source: PLoS One. 2023 Oct 19;18(10):e0292893. doi: 10.1371/journal.pone.0292893 (PMC10586667; doi:10.1371/journal.pone.0292893)
Supplement: Attachment — Submitted filename: Plos-one-comments-resubmission.docx [file pone.0292893.s003.docx]

I am happy to accept the manuscript (although I include very minor revision notes below). Please note I am referring to the manuscript I was sent directly by the editor and not the version I could download either via the portal or the link in the e-mail.

The manuscript was much improved with regards to clarity and my concerns were addressed. I feel the rewording allowed the novelty and main findings of the work to be expressed more clearly.

On the manuscript I was sent with changes tracked I spotted 3 very minor errors.

On Line 944 The authors (as in the editor notes) need to clarify what A,B etc are

On Line 1283 The authors refer to the PWN as B. xylophilus for the first time. This should be the full latin name and in the introduction not the discussion.

In the discussion the numbering has broken down as (1) is deleted. Therefore (2) on line 164 needs editing (or removing).
